# Complications leading to hospitalisation 12 months after brachytherapy or high-intensity focused ultrasound for localized prostate cancer: French national from the PMSI-MCO data, 2019 and 2020

**DOI:** 10.1016/j.ctro.2024.100854

**Published:** 2024-09-12

**Authors:** Timothée Bourgarit, Clément Larose, Andrea Dagry, Nicolas Martz, Beverley Balkau, Pascal Eschwège, Charles Mazeaud

**Affiliations:** a Department of Urology CHRU Nancy, Brabois Hospital, 54000 Nancy, France; b Real Consulting Data, 92120 Montrouge, France; c Radiation Department, Lorraine Cancer Institute, 54000 Nancy, France; d INSERM U1018, Clinical Epidemiology, CESP, 94807 Villejuif, France; e CNRS UMR 7039, Tumor Biology Unit, CRAN, 54547 Vandœuvre-lès-Nancy, France; f Université de Lorraine, Inserm, IADI U1254, 54000 Nancy, France

**Keywords:** Brachytherapy, Complications, HIFU, Localized prostate cancer

## Abstract

**Background and purpose:**

Prostate cancer can be treated using either brachytherapy or high-intensity focused ultrasound (HIFU), which are less invasive than surgery. Although both approaches have proved effective, few studies have looked at the specific causes of hospitalisation due to complications, following these treatments. The aim of this study was to compare the causes of hospitalisation.

**Methods:**

A retrospective study was carried out examining the records of patients who had undergone brachytherapy or HIFU treatment for localized prostate cancer in 2019 and 2020, using the French national database: Programme de Médicalisation du Système d’Information − Médecine, Chirurgie, Obstétrique (PMSI-MSO). Data on post-treatment hospitalisations were analyzed.

**Results:**

3090 patients were included in the study, of whom 1699 underwent brachytherapy and 1391 HIFU procedures. The incidence of hospitalisation was much higher after HIFU than after brachytherapy, notably due to a higher rate of obstructive complications (12.94% vs 2.77%). Large differences were also found for infections (8.20% vs 1.47%) and bleeding (6.76% vs 2.18%) leading to hospitalisation. Most of the complications occurred at the initial hospitalization: 12% for HIFU, and 1.4% for brachytherapy.

**Conclusion:**

Complications were more frequent after treatment with HIFU than with brachytherapy in the year following treatment for localized prostate cancer. Further the causes of hospitalisation differed between the two treatments. These differences need to be taken into account in the therapeutic strategy, as well as in post-treatment management.

## Highlights

• This is the first study to report real life data on hospitalisation after treatment with brachytherapy or HIFU, using French national PMSMCO data over two consecutive years.
• In 2019 and 2020, 1699 patients underwent brachytherapy and 1391 HIFU procedures respectively.
• Hospitalization was more frequent after HIFU, mainly due to more obstructive complications (12,94% vs 2.77%).
• HIFU led to more infections (8.20% vs 1.47%) and bleeding (6.76% vs 2.18%) compared to brachytherapy.
• Patients must be informed of potential complications of brachytherapy and HIFU when choosing their treatment.

## Introduction

Prostate cancer has seen an increase in incidence with the development of individual screening, leading to a significant reduction in mortality. In France in 2015, there were 50,400 new cases of diagnosed prostate cancer [1]. The evolution over time and the development of prostate-specific antigen (PSA) testing since the 1990 s have permitted early and localized detection of prostate cancer, enabling treatment options, both surgical and non-surgical, as per current guidelines [2]. Several other alternatives are still under study, in particular focal treatments for prostate cancer, but they are not yet included in treatment recommendations. Therapeutic strategy is based on the pathology grade of the prostate cancer, clinical and imaging data, age, patient history, and preferences.

Depending on the therapy, complications may arise in the short or long term. These complications can vary in nature and include infectious, hemorrhagic, thromboembolic, and functional issues. Some complications, such as erectile dysfunction and urinary incontinence, can be managed through consultations, while others may require hospitalisation. This study focuses on complications that led to hospitalisation.

Given the multiple treatment modalities available for localized prostate cancer, it is essential to thoroughly understand the complications associated with these treatments and their timing. This knowledge will help develop appropriate therapeutic strategies and provide patients with clear information about the risks of their chosen treatment.

In this retrospective epidemiological study, we looked at brachytherapy and HIFU (High-Intensity Focalised Ultrasound), two validated treatments as an alternative to the standard of care (external radiotherapy or surgery). These two therapeutic strategies have the same indication in the treatment of low- or intermediate-risk localised prostate cancer, with a good prognosis. Treatment with LDR brachytherapy is now very well established and has clear protocols. However, this needs to be clarified for HIFU treatments, which have variable oncological and functional post-treatment results from one study to another. The European Association of Urology (EAU) and the American Urological Association (AUA) describe a lack of perspective and comparable data on HIFU treatment modalities [3], [4].

The aim is to analyse the incidence of complications leading to hospitalisation in the year following treatment with brachytherapy and with HIFU.

## Methods and material

### The PMSI-MCO

Since the implementation of “fee-for-service” in 2005, healthcare facilities in France must use the Programme de Médicalisation du Système d'Information − Médecine, Chirurgie, Obstétrique (PMSI-MSO) to receive budget allocations corresponding to their activities. This mandatory system provides information on diagnoses, medical procedures performed, and hospital stays. Each medical procedure and diagnosis is coded using the International Classification of Diseases, 10th revision (ICD-10) for diagnoses and the Common Classification of Medical Acts (CCAM) for procedures. This classification defines reimbursement for the French national healthcare insurance system.

Ethical approval was not required for using this aggregated data; all information was anonymous.

#### Study population

The study population was selected from the national PMSI-MCO database and included all patients with a diagnosis of non-metastatic prostate cancer in 2019 and 2020 with procedures performed, based on their CCAM codes:

− Brachytherapy LDR (Low Dose Rate) with permanent insertion of iodine 125 with CCAM code JGNL001

− HIFU (High Intensity Focalised Ultrasound) with CCAM code JGNJ900

#### Complications

Complications leading to hospitalisation during the year following treatment were analyzed for the two procedures. The PMSI-MCO only collects data from hospitalisations. Complications were coded according to the ICD-10 classification from the standardized discharge summaries (RSS), classified as the primary or associated diagnoses. Complications were grouped into the ten categories shown in Supplementary Table 1. For reasons of confidentiality, these data are only available when there are more than ten individuals in any category.

#### Timing of hospitalisations

We analysed the incidence of these complications leading to hospitalization in the year following the procedure: at the initial hospitalisation, T0; during the first month, M1; during months 2 to 6, M2-M6; during months 7–12, M7-M12.

#### Data processing

All data were processed using Microsoft Excel spreadsheet software. These are global real-life data from the French population, not a population sample. The differences in the percentages of complications were calculated, and those over 1 % have been highlighted by bold text in Table 1. The differences observed were not due to chance, so no statistical tests were applied in our study.

**Table 1:** Complications leading to hospitalisation following treatment by brachytherapy or high-intensity focused ultrasound (HIFU) for localized prostate cancer, with the corresponding ICD-10 codes: raw and percentage data for 2019 and 2020 for the year following treatment. Cells highlighted in bold show where there were differences greater than 1% between procedures. French national data from the PMSI-MCO data base. There is only the complications for which an incidence was observed are included in this table. The full list of complication leading to hospitalization is provided in Supplementary Table 1.

**Complications, over the 12 months after treatment**	**ICD-10 codes**	**Brachytherapy (JGNL001)**				**HIFU (JDNJ900)**			**Difference: Brachytherapy – HIFU** **2019 and 2020**
		**2019**	**2020**	**2019 and 2020**		**2019**	**2020**	**2019 and 2020**	
		**n = 968**	**n = 731**	**n = 1699**		**n = 795**	**n = 596**	**n = 1391**	
**Infectious complications**		25 (2.58 %)	0	25 (1.47 %)		66 (8.30 %)	48 (8.05 %)	114 (8.20 %)	**−6.72 %**
Inflammatory diseases of prostate	N41	14 (1.45 %)	0	14 (0.82 %)		32 (4.03 %)	36 (6.04 %)	68 (4.89 %)	**−4.06 %**
Orchitis and epididymitis	N45	0	0	0		11 (1.38 %)	0	11 (0.79 %)	−0.79 %
Urinary tract infection	N39.0	11 (1.14 %)	0	11 (0.65 %)		23 (2.89 %)	12 (2.01 %)	35 (2.52 %)	**−1.87 %**
**Bleeding complications**		18 (1.86 %)	19 (2.60 %)	37 (2.18 %)		48 (6.04 %)	46 (7.72 %)	94 (6.76 %)	**−4.58 %**
Hemorrhage or hematoma complicating a procedure	T81.0	0	0	0		27 (3.40 %)	21 (3.52 %)	48 (3.45 %)	**−3.45**
Hematuria	R31	18 (1.86 %)	19 (2.60 %)	37 (2.18 %)		21 (2.64 %)	25 (4.19 %)	46 (3.31 %)	**−1.13 %**
**Acute obstructive complications**		24 (2.48 %)	23 (3.15 %)	47 (2.77 %)		113 (14.21 %)	67 (11.24 %)	180 (12.94 %)	**−10.17 %**
Retention of urine	R33	24 (2.48 %)	23 (3.15 %)	47 (2.77 %)		113 (14.21 %)	67 (11.24 %)	180 (12.94 %)	**−10.17 %**
**Bladder complications**		0	0	0		36 (4.53 %)	14 (2.35 %)	50 (3.59 %)	**−3.59 %**
Bladder-neck obstruction	N32.0	0	0	0		25 (3.14 %)	14 (2.35 %)	39 (2.80 %)	**−2.80 %**
Calculus of lower urinary tract	N21	0	0	0		11 (1.38 %)	0	11 (0.79 %)	−0.79 %
**Urethral complications**		0	0	0		21 (2.64 %)	0	21 (1.51 %)	**−1.51 %**
Urethral stricture	N35	0	0	0		21(2.64 %)	0	21 (1.51 %)	**−1.51 %**
**Functional complications**		0	0	0		17 (2.14 %)	0	17 (1.22 %)	**−1.22 %**
Dysuria	R30.0	0	0	0		17 (2.14 %)	0	17 (1.22 %)	**−1.22 %**

## Results

### Patient groups and baseline characteristics

In 2019, 968 brachytherapy and 795 HIFU treatments were carried out for localised prostate cancer. In 2020, there were fewer treatments using these methods, with 731 brachytherapy and 596 HIFU (Table 2).

**Table 2:** Characteristics [n (%)] of men treated by brachytherapy or high-intensity focused ultrasound (HIFU) for localized prostate cancer in 2019 and 2020. French national data from the PMSI-MCO database.

	ICD-10 codes	Brachytherapy (JGNL001)		HIFU (JGNJ900)	
		2019n = 968	2020n = 731	2019n = 795	2020n = 596
Age > 60 years		823 (85 %)	611 (84 %)	752 (95 %)	573 (96 %)
Diabetes	E11	24 (2.5 %)	15 (2.1 %)	46 (5.8 %)	27 (4.5 %)
Hypertension	I10	59 (6.1 %)	38 (5.2 %)	115(14.5 %)	52(8.7 %)
Anticoagulant treatment	Z92.1	11 (1.1 %)	0	41 (5.2 %)	16 (2.7 %)
Obesity	E66	31 (3.2 %)	30 (4.1 %)	25 (3.1 %)	19 (3.2 %)

Patients treated with brachytherapy were younger, fewer had diabetes, were hypertensive or treated with anticoagulants. There were similar rates of obesity.

### Incidence of complications in the year following brachytherapy treatment

We found very few complications leading to hospitalization after brachytherapy: 1.47 % were infectious (inflammatory diseases of the prostrate or urinary tract infections); 2.18 % were bleeding (hematuria); 2.77 % acute obstructive (retention of urine) (Table 1).

**Fig. 1:**
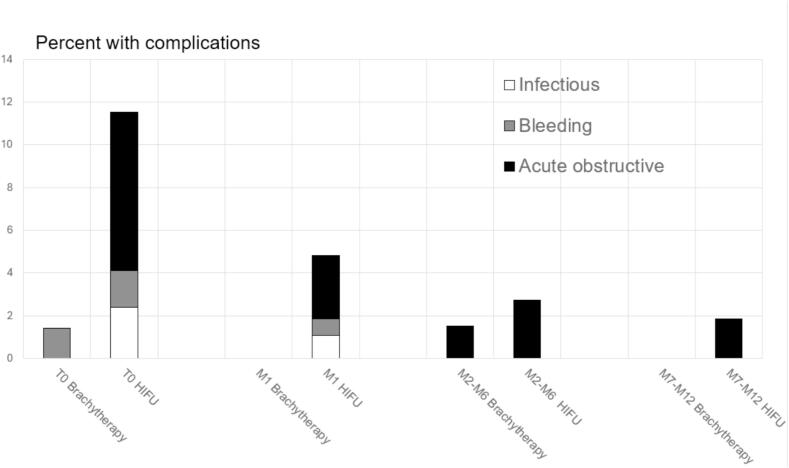
Percentage of patients with complications leading to hospitalisation over periods within one year of treatment after brachytherapy and high-intensity focused ultrasound (HIFU) in patients with localised prostate cancer. Data from the French PMSI-MCO. Initial hospitalisation, Time 0 (T0); during the first month, Month 1(M1); during months 2 to 6, M2-M6; during months 7–12, M7-M12.

The timing of these complications was able to be determined only if there were more than 10 recorded in a category (confidentiality reasons). At T0 the only complications recorded were hematuria in 1.41 % of patients (Fig. 1). Between the 2nd and 6th months following treatment, hospitalisation for acute urine retention was recorded in 1.53 % of patients.

### Incidence of complications in the year following treatment with HIFU

The most frequent complications were acute obstructive (retention of urine), 12.94 %, followed by infections (inflammatory disease of the prostrate, orchitis and epididymitis, urinary tract infection) 8.20 %, then by bleeding (hematoma, hematuria) 6.76 %, bladder (bladder-neck constriction, calculus of lower urinary tract) 3.59 %. The frequencies of urethral and functional complications were lower.

For the timing of these complications, obstructive complications (retention of urine) were recorded at all time intervals, with the frequency reducing over time, from 7.40 % to 1.87 % during the last 6 months of follow-up (Fig. 1). We note incidences of bleeding complications during the initial stay in 1.73 % of patients and during the first month, 0.79 %. Infectious complications occurred in 2.40 % of patients during their initial stay, 1.08 % in the following month.

### Comparison of the two treatments

Comparing the results over the entire twelve months following treatments in 2019 and 2020, differences greater than 1 % are highlighted by bold text in Table 1. There were fewer infectious complications after brachytherapy, with a difference of 6.72 %, due to differences for “inflammatory diseases of prostate” and the “urinary tract infection”.

There were also fewer bleeding complications with brachytherapy, a difference of 4.58 % with fewer hospitalisations for “haematuria” and for the coding “Haemorrhage or haematoma complicating a procedure”.

For brachytherapy there were 10.17 % fewer acute urine retention episodes, and 3.59 % fewer bladder complications, essentially due to obstructions of the bladder neck for patients treated by brachytherapy.

Other complications point in the same direction: there was less dysuria and urethral stricture after treatment with brachytherapy than with HIFU.

## Discussion

We noted a slight decrease in the number of diagnoses and treatments between the 2019 and 2020 data, which can be linked to the COVID-19 health crisis and the measures taken at the time [5].

### Brachytherapy

Our study shows that there were very few complications leading to hospitalisation after brachytherapy treatment. Infectious complications occurred in 1.47 % of patients, haemorrhagic complications such as haematuria in 2.18 % and acute urine retention in 2.77 %.

In this study, we have focused exclusively on LDR brachytherapy, considering only the CCAM coding for the permanent implantation of iodine-125 seeds. HDR (High Dose Rate) brachytherapy was occasionally performed in expert centers in France for intermediate—or high-risk localized prostate cancers. It was often combined with external radiotherapy to achieve a 'boost' effect, though it was associated with more significant side effects [6].

The development of techniques for implanting iodine 125 seeds in the periphery of the prostate has led to an improvement in the morbidity of brachytherapy, particularly in terms of incontinence. This is another reason why this treatment method should be carried out in expert centres, to minimise the adverse effects associated with the implantation technique [7].

A review of the literature, published in 2018, estimated the risk of acute urine retention at between 2 and 10 % and the risk of infectious complications at less than 5 % [8]. The studies report prostate volume and the International Prostate Symptom Score (IPSS) as factors predictive of acute urine retention and also for judging the cessation of a prostatic desobstruction procedure [8], [9]. These parameters should therefore be taken into account when choosing brachytherapy as a treatment.

Brachytherapy is therefore a treatment with good tolerability if it is carried out for the right indications and in expert centres. The long-term rehospitalisation rates found in the literature are also low [9], [10].

A study by Hunter et al. found a 4.3 % cumulative incidence of late genitourinary toxicity >=2(according to the Radiation Therapy Oncology Group criteria) with no genitourinary toxicity beyond the first four years, thus probably no hospitalisation in the 4 to 10 years following treatment [11].

### High-intensity focused ultrasound (HIFU)

All complications were more frequent after the HIFU procedure.

Our study found a high incidence of infectious (8.20 %), bleeding (6.76 %) and acute obstructive (12.94 %) complications with HIFU. This is the treatment with the higher incidence of acute obstructive complications, due to episodes of acute retention of urine. Other complications included bladder neck obstructions (2.58 % of patients) and urethral strictures (1.51 % of patients).

A major study carried out in Lyon, France and published in 2014 on 1002 patients reported results at 5 years [12]. This study found an incontinence rate of around 18 % and an obstructive complication rate of 26 % (urine retention, stenosis).

A review of the literature on HIFU treatment, published in 2022, had complications reported in 13 studies [13]. Acute retention of urine following the procedure was reported in eight of these studies and ranged from 7 to 27 %, mostly early post-operative, complications up to 3 months after the procedure. This was in line with our data of 13 %.

Patients treated with HIFU tend to be older and have higher morbidity (Table 2); However, the treatment specific complications were more frequent compared to brachytherapy. Our study provided insight into the potential complications of HIFU in a real-world population, challenging the previous perception of HIFU as a non-invasive treatment option.

### Strengths and limitations

Our study is a retrospective study based on PMSI-MCO data. It is therefore very powerful because it is compulsory for all hospitals to provide this data. These are real-life data. However, it is limited in that if there are fewer than 10 observations in a particular cross classification, this data is not available for analysis and is set to zero, for reasons of patient confidentiality. This limits the analysis according to the timing of the complications.

PMSI-MCO data are both our main advantage and our limitation, given the medico-economic nature of this data, which can lead to coding biases [14].

To avoid our cohort being a prostate cancer re-treatment, patients were diagnosed with localized prostate cancer in 2019 or 2020 and underwent treatment with HIFU or brachytherapy in the same year.

Treatment of the entire prostate gland is associated with more genitourinary complications than focal treatment [15]. One bias in our study was the impossibility of knowing whether the treatment involved the whole prostate gland or only a focal treatment. There is only one CCAM code for brachytherapy or HIFU, which does not differentiate between complete prostate treatment and focal treatment.

The information gathered from inpatient diagnoses is limited, so we could not obtain data concerning, for example, the anatomopathological nature of patients treated, the size of the prostate, the treated target volume, or biological recurrences. This information only gives data concerning hospitalisation. We do not know the incidence of complications (continence, erectile function) that are managed on an outpatient or inpatient basis.

### Perspectives

It would be interesting to carry out a follow-up of more than one year to analyse, using PMSI-MCO data, the complications that we know occur later (urodigestive fistula, urethral stenosis, ureteral stenosis, haematuria due to radiation cystitis, etc.).

A more complete linkage with other French databases (CepiDC, SNIIRAM) would be interesting in order to have a more complete database in terms of consumption of care and complications linked to the treatment carried out.

## Conclusion

HIFU leads to a much higher number of re-hospitalizations for complications than curietherapy. Even if these two minimally invasive therapies are difficult to compare, our general population data argue in favor of more studies on the side effects of HIFU. When informing patients, it is essential to explain the specific risks of these two treatments.

## Declaration of competing interest

The authors declare that they have no known competing financial interests or personal relationships that could have appeared to influence the work reported in this paper.
