# Supplementary material for: Complications leading to hospitalisation 12 months after brachytherapy or high-intensity focused ultrasound for localized prostate cancer: French national from the PMSI-MCO data, 2019 and 2020
Source: Clin Transl Radiat Oncol. 2024 Sep 12;49:100854. doi: 10.1016/j.ctro.2024.100854 (PMC11459057; doi:10.1016/j.ctro.2024.100854)
Supplement: Supplementary Data 1 [file mmc1.docx]

| **Complications, over the 12 months after treatment** | **ICD-10**  **codes** | **Brachytherapy (JGNL001)** | | |  | **HIFU (JDNJ900)** | | | **Difference: Brachytherapy – HIFU**  **2019 and 2020** |
| --- | --- | --- | --- | --- | --- | --- | --- | --- | --- |
|  |  | **2019** | **2020** | **2019 and 2020** |  | **2019** | **2020** | **2019 and 2020** |  |
|  |  | **n=968** | **n=731** | **n=1699** |  | **n=795** | **n=596** | **n=1391** |  |
| **Infectious/Inflammatory complications** |  | 25 (2.58%) | 0 | 25 (1.47%) |  | 66 (8.30%) | 48 (8.05%) | 114 (8.20%) | **-6.72%** |
| Inflammatory diseases of prostate | N41 | 14 (1.45%) | 0 | 14 (0.82%) |  | 32 (4.03%) | 36 (6.04%) | 68 (4.89%) | **-4.06%** |
| Orchitis and epididymitis | N45 | 0 | 0 | 0 |  | 11 (1.38%) | 0 | 11 (0.79%) | -0.79% |
| Sepsis | A41 | 0 | 0 | 0 |  | 0 | 0 | 0 | - |
| Urinary tract infection | N39.0 | 11 (1.14%) | 0 | 11 (0.65%) |  | 23 (2.89%) | 12 (2.01%) | 35 (2.52%) | **-1.87%** |
| Cystitis | N30 | 0 | 0 | 0 |  | 0 | 0 | 0 | - |
| Septic shock | R57.2 | 0 | 0 | 0 |  | 0 | 0 | 0 | - |
| Fever of undetermined origin | R50 | 0 | 0 | 0 |  | 0 | 0 | 0 | - |
| Peritonitis | K65 | 0 | 0 | 0 |  | 0 | 0 | 0 | - |
| **Bleeding complications** |  | 18 (1.86%) | 19 (2.60%) | 37 (2.18%) |  | 48 (6.04%) | 46 (7.72%) | 94 (6.76%) | **-4.58%** |
| Hemorrhage | R58 | 0 | 0 | 0 |  | 0 | 0 | 0 | - |
| Acute posthemorrhagic anemia | D62 | 0 | 0 | 0 |  | 0 | 0 | 0 | - |
| Hemorrhage or hematoma complicating a procedure | T81.0 | 0 | 0 | 0 |  | 27 (3.40%) | 21 (3.52%) | 48 (3.45%) | **-3.45** |
| Iron deficiency anemia secondary to blood loss (chronic) | D50.0 | 0 | 0 | 0 |  | 0 | 0 | 0 | - |
| Hematuria | R31 | 18 (1.86%) | 19 (2.60%) | 37 (2.18%) |  | 21 (2.64%) | 25 (4.19%) | 46 (3.31%) | **-1.13%** |
| Hypovolemic shock | R57.1 | 0 | 0 | 0 |  | 0 | 0 | 0 | - |
| Volume depletion | E86 | 0 | 0 | 0 |  | 0 | 0 | 0 | - |
| Recurrent and persistent hematuria | N02 | 0 | 0 | 0 |  | 0 | 0 | 0 | - |
| **Thrombo-embolic complications** |  | 0 | 0 | 0 |  | 0 | 0 | 0 |  |
| Pulmonary embolism | I26 | 0 | 0 | 0 |  | 0 | 0 | 0 | - |
| Phlebitis and thrombophlebitis | I80 | 0 | 0 | 0 |  | 0 | 0 | 0 | - |
| Other venous embolism and thrombosis | I82 | 0 | 0 | 0 |  | 0 | 0 | 0 | - |

Supplementary Table 1: Complications leading to hospitalisation following treatment by brachytherapy or high-intensity focused ultrasound (HIFU) for localized prostate cancer, with the corresponding ICD-10 codes: raw and percentage data for 2019 and 2020 for the year following treatment. Cells highlighted in bold show where there were differences greater than 1% between procedures. French national data from the PMSI-MCO data base.

| **Acute obstructive complications** |  | 24 (2.48%) | 23 (3.15%) | 47 (2.77%) |  | 113 (14.21%) | 67 (11.24%) | 180 (12.94%) | **-10.17%** |
| --- | --- | --- | --- | --- | --- | --- | --- | --- | --- |
| Retention of urine | R33 | 24 (2.48%) | 23 (3.15%) | 47 (2.77%) |  | 113 (14.21%) | 67 (11.24%) | 180 (12.94%) | **-10.17%** |
| Obstructive and reflux uropathy | N13 | 0 | 0 | 0 |  | 0 | 0 | 0 | - |
| Acute renal failure | N17 | 0 | 0 | 0 |  | 0 | 0 | 0 | - |
| Anuria and oliguria | R34 | 0 | 0 | 0 |  | 0 | 0 | 0 | - |
| **Bladder complications** |  | 0 | 0 | 0 |  | 36 (4.53%) | 14 (2.35%) | 50 (3.59%) | **-3.59%** |
| Injury of bladder | S37.2 | 0 | 0 | 0 |  | 0 | 0 |  |  |
| Vesical fistula | N32.2 | 0 | 0 | 0 |  | 0 | 0 |  |  |
| Bladder-neck obstruction | N32.0 | 0 | 0 | 0 |  | 25 (3.14%) | 14 (2.35%) | 39 (2.80%) | **-2.80%** |
| Accidental puncture and laceration during procedure | T81.2 | 0 | 0 | 0 |  | 0 | 0 | 0 | - |
| Calculus of lower urinary tract | N21 | 0 | 0 | 0 |  | 11 (1.38%) | 0 | 11 (0.79%) | --0.79% |
| **Urethral complications** |  | 0 | 0 | 0 |  | 21 (2.64%) | 0 | 21 (1.51%) | **-1.51%** |
| Postprocedural urethral stricture | N99.1 | 0 | 0 | 0 |  | 0 | 0 | 0 | - |
| Injury of urethra | S37.3 | 0 | 0 | 0 |  | 0 | 0 | 0 |  |
| Urethral fistula | N36.0 | 0 | 0 | 0 |  | 0 | 0 | 0 | - |
| Other specified disorders of urethra | N36.8 | 0 | 0 | 0 |  | 0 | 0 | 0 | - |
| Urethral stricture | N35 | 0 | 0 | 0 |  | 21(2.64%) | 0 | 21 (1.51%) | **-1.51%** |
| Urethritis and urethral syndrome | N34 | 0 | 0 | 0 |  | 0 | 0 | 0 | - |
| **Ureteral complications** |  | 0 | 0 | 0 |  | 0 | 0 | 0 | - |
| Other specified disorders of kidney and urether | N28.8 | 0 | 0 | 0 |  | 0 | 0 | 0 | - |
| **Functional complications** |  | 0 | 0 | 0 |  | 17 (2.14%) | 0 | 17 (1.22%) | **-1.22%** |
| Unspecified urinary incontinence | R32 | 0 | 0 | 0 |  | 0 | 0 | 0 |  |
| Dysuria | R30.0 | 0 | 0 | 0 |  | 17 (2.14%) | 0 | 17 (1.22%) | **-1.22%** |
| Other specified urinary incontinence | N39.4 | 0 | 0 | 0 |  | 0 | 0 | 0 | - |
| Stress incontinence | N39.3 | 0 | 0 | 0 |  | 0 | 0 | 0 | - |
| Neuromuscular dysfunction of bladder | N31 | 0 | 0 | 0 |  | 0 | 0 | 0 | - |
| Fecal incontinence | R15 | 0 | 0 | 0 |  | 0 | 0 | 0 | - |
| **Rectal complications** |  | 0 | 0 | 0 |  | 0 | 0 | 0 | - |
| Injury of rectum | S36.6 | 0 | 0 | 0 |  | 0 | 0 | 0 | - |
| Radiation sickness | T66 | 0 | 0 | 0 |  | 0 | 0 | 0 | - |
| **Complications associated with radiotherapy** |  | 0 | 0 | 0 |  | 0 | 0 | 0 | - |
| Radiation sickness | T66 | 0 | 0 | 0 |  | 0 | 0 | 0 | - |
| Other and unspecified dermatitis | L30 | 0 | 0 | 0 |  | 0 | 0 | 0 | - |
